# CausalMGM: an interactive web-based causal discovery tool

**DOI:** 10.1093/nar/gkaa350

**Published:** 2020-05-11

**Authors:** Xiaoyu Ge, Vineet K Raghu, Panos K Chrysanthis, Panayiotis V Benos

**Affiliations:** Department of Computer Science, University of Pittsburgh, 4200 Fifth Avenue, Pittsburgh, PA 15260, USA; Department of Computer Science, University of Pittsburgh, 4200 Fifth Avenue, Pittsburgh, PA 15260, USA; Department of Computational and Systems Biology, University of Pittsburgh, 3420 Forbes Ave, Pittsburgh, PA 15213, USA; Department of Computer Science, University of Pittsburgh, 4200 Fifth Avenue, Pittsburgh, PA 15260, USA; Department of Computer Science, University of Pittsburgh, 4200 Fifth Avenue, Pittsburgh, PA 15260, USA; Department of Computational and Systems Biology, University of Pittsburgh, 3420 Forbes Ave, Pittsburgh, PA 15213, USA

## Abstract

High-throughput sequencing and the availability of large online data repositories (e.g. The Cancer Genome Atlas and Trans-Omics for Precision Medicine) have the potential to revolutionize systems biology by enabling researchers to study interactions between data from different modalities (i.e. genetic, genomic, clinical, behavioral, etc.). Currently, data mining and statistical approaches are confined to identifying correlates in these datasets, but researchers are often interested in identifying cause-and-effect relationships. Causal discovery methods were developed to infer such cause-and-effect relationships from observational data. Though these algorithms have had demonstrated successes in several biomedical applications, they are difficult to use for non-experts. So, there is a need for web-based tools to make causal discovery methods accessible. Here, we present CausalMGM (http://causalmgm.org/), the first web-based causal discovery tool that enables researchers to find cause-and-effect relationships from observational data. Web-based CausalMGM consists of three data analysis tools: (i) feature selection and clustering; (ii) automated identification of cause-and-effect relationships via a graphical model; and (iii) interactive visualization of the learned causal (directed) graph. We demonstrate how CausalMGM enables an end-to-end exploratory analysis of biomedical datasets, giving researchers a clearer picture of its capabilities.

## INTRODUCTION

One of the primary goals of biomedical research is to understand the etiology of chronic disease and disease progression. Recent technological advances such as next-generation sequencing (1) and the ubiquity of sensors (e.g. smartphones, smartwatches, etc.) (2) have provided us with large multi-modal databases capable of improving our understanding of chronic disease. However, two main challenges prevent the usage of common data analysis tools on these datasets. First, most of these datasets are observational, so techniques that can automatically identify cause-and-effect relationships from observational data are required. Second, these datasets contain mixed data types (i.e. continuous and discrete variables). Most common analysis techniques such as machine learning (3) and correlation networks (4) are ill-suited for these challenges since they focus upon correlations that overestimate the number of causal associations and since they typically operate on datasets with only a single variable type.

Causal discovery methods offer a promising solution. These methods take observational data as input and they output a graph where nodes correspond to variables in the data and edges correspond to direct (causal) relationships (5). The most popular of these is PC (6), a constraint-based algorithm, which starts with a fully connected graph and uses conditional independence tests to prune the space of causal graphs consistent with the observed data. Recently, we and others have extended causal discovery methods to operate on mixed datasets (with continuous and categorical data) (7–10) and these novel methods have demonstrated successes on biomedical applications (7,11–13). Despite this, causal discovery algorithms are available for public use only via desktop applications (14) and programmatic interfaces (https://bd2kccd.github.io/docs/causal-cmd/), with a notable exception of one recent web application that constructs a causal graph from (continuous) single-cell flow cytometry data (15).

**Figure 1.**
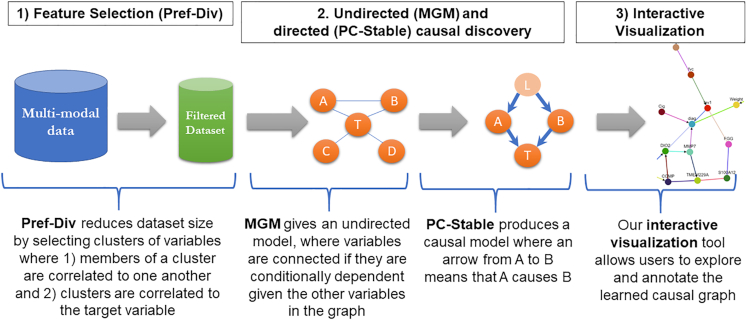
CausalMGM analysis framework. The server takes a tabular, multi-modal dataset as input and performs (i) feature selection and clustering, (ii) undirected and directed causal discovery, and (iii) interactive visualization of the learned causal graph.

To this end, we developed CausalMGM, a web-based causal discovery tool for mixed datasets. CausalMGM is a suite of tools for causal discovery and visualization (Figure 1) that takes a tabular, observational dataset as input and performs three sequential operations: (i) informative pre-selection and clustering of features to model using Preferential Diversity (Pref-Div) (16–18); (ii) causal discovery from mixed data using a two-step approach we developed (7,19); and (iii) interactive visualization of the learned causal graph. Altogether, this process enables end-to-end causal discovery from observational, mixed data. The server is freely available to the public for non-commercial use and licensed under Creative Commons Attribution-NonCommercial 4.0 International License (20). The remainder of this paper presents the server in detail and is organized as follows:

- First, we present the implementation details of the web server.
- Next, we present the feature selection method, Pref-Div, and the causal discovery methods used in greater detail.
- Then, we demonstrate how the CausalMGM server can be used on real data and discuss the proper interpretation of the results.
- Finally, we discuss the limitations of the server and potential future directions.

## METHODS AND IMPLEMENTATION

In this section, we give details about the how the web server was implemented and summarize each of the computational methods underlying the full workflow.

### The CausalMGM web server

**Figure 2.**
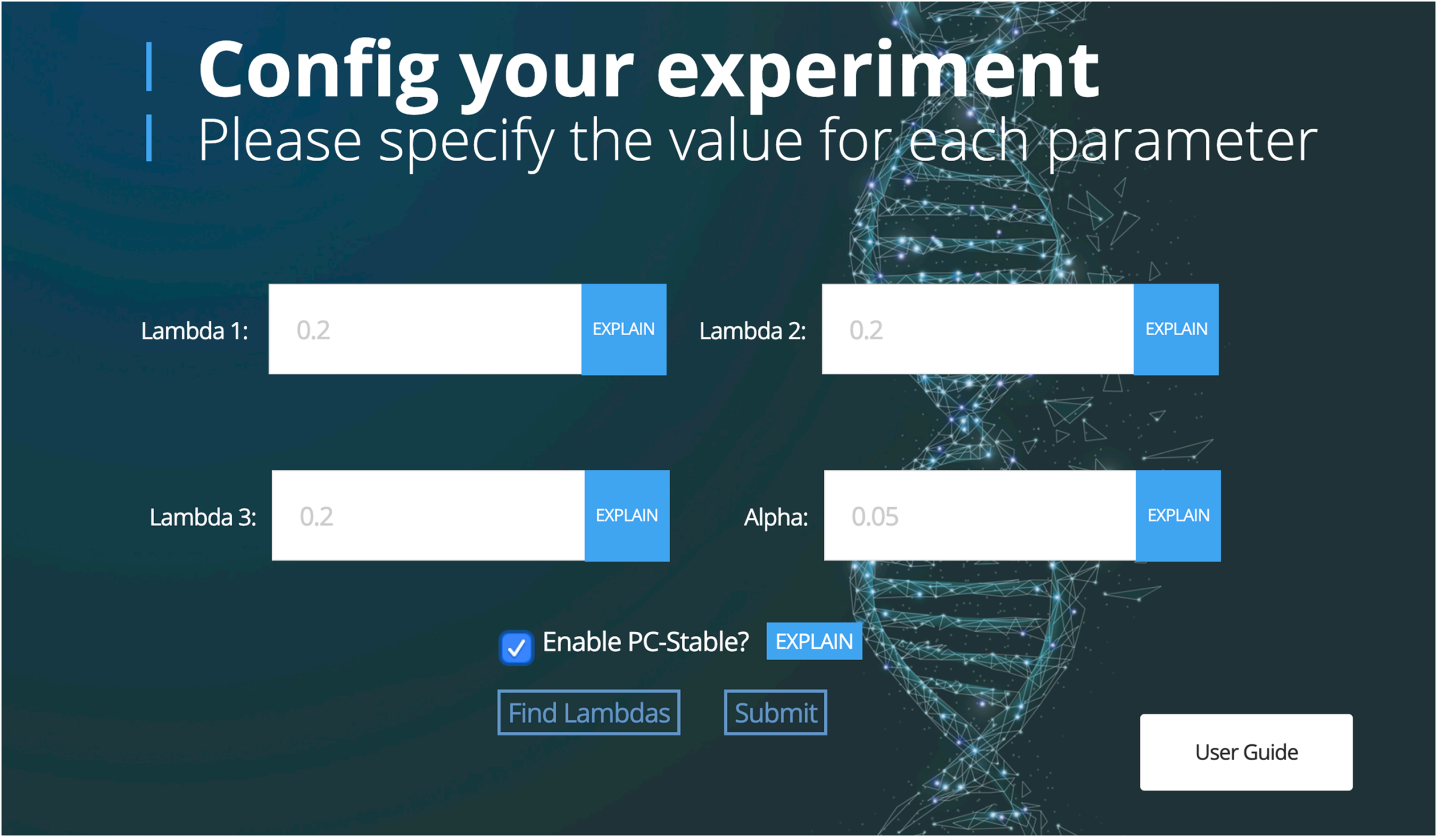
Example of CausalMGM web server user interface.

Figure 1 illustrates the overall workflow of the CausalMGM web server, including feature selection, undirected and directed causal discovery, and interactive visualization. CausalMGM consists of a user-friendly interface (e.g. Figure 2) and is ideal for non-advanced users. Leveraging the state-of-the-art algorithms, mixed graphical models (MGM) (7) and PC-Stable (21), the CausalMGM web server enables researchers to find both conditional dependencies and causal relationships between features of an observational, biomedical dataset. In addition, researchers have the option to perform automatic pre-selection of features (i.e. dimensionality reduction) of their data to a customizable lower dimensional subset.

The back end of the CausalMGM web server is implemented using Java 8, and the front end is constructed from JavaScript, jQuery and PHP. The interactive visualization is developed using the popular open-source library Cytoscape.js (22), which allows the CausalMGM web server to produce dynamic online visualizations that are compatible with the Cytoscape desktop application. Next, we will discuss in depth each of the main components of the CausalMGM web server.

### Feature selection: Preferential Diversity

Since causal discovery methods are generally inefficient on large datasets, we provide a method for feature pre-selection and clustering: Pref-Div (16), to help users focusing on the variables that are more likely to yield meaningful causal networks. The inputs to Pref-Div are (i) a tabular dataset, (ii) a target variable of interest, (iii) the total number of variables to select and (iv) whether automatic clustering should be performed (yes/no). The main idea of Pref-Div is to identify variables that are associated with the target variable of interest but are maximally independent of one another, similarly to principal component analysis. Pref-Div is an iterative algorithm that first chooses *k* variables most associated with the target and sorts them by strength of association. It then iterates through these *k* variables and ‘marks’ those that are closely associated with a variable already selected. If automated clustering is included, then these ‘marked’ variables are included in the final result as part of a cluster, otherwise they are removed. The other variables in the top *k* are included in the final result set. This process is repeated until the number of variables selected equals the number of variables requested by the user.

In the current web server, when the target variable is continuous, Pearson correlation is used as a measure of association between the target variable and the query variable *X*. When the target is categorical, we use (1 − *p*), where *p* is the *P*-value from a likelihood ratio test between a null model and a logistic regression model with *X* as a predictor. The correlation threshold to determine whether two variables should be ‘marked’ or clustered is determined based upon stability (insensitivity to small variations in the data) (17). Currently, categorical variables are automatically included in the final result, and the user can optionally choose continuous variables to keep as well. The use of this procedure is optional, especially when datasets are relatively small (<100 variables).

### Causal discovery: MGM PC-Stable

The next step in our pipeline produces a causal graph from the resulting filtered dataset. The causal discovery algorithm implemented in the CausalMGM web server (MGM PC-Stable) first learns an undirected graph using our MGM method and then uses it as skeleton to learn the causal directions (PC-Stable) (7). We first discuss learning an undirected model using MGM and an optional step to automatically select the regularization parameters using the StEPS (stable edge-specific penalty selection) procedure (19). Then, we discuss learning the directed model structure using PC-Stable on the undirected graph (7). Finally, we present our independence test for mixed datasets.

#### MGM

MGM expects a tabular dataset as input and it outputs a graph, where nodes correspond to variables and edges correspond to conditional dependencies. A (undirected) edge between two variables *A* and *B* implies that *A* and *B* are dependent conditioned on the rest of the variables in the dataset. The algorithm finds the optimal undirected graph by optimizing the pseudo-likelihood of the data given the model using a gradient-based procedure (proximal gradient). To ensure a sparse graph, the pseudo-likelihood is subject to sparsity penalties (*λ*_CC_, *λ*_CD_, *λ*_DD_), where CC is the regularization parameter for edges between two continuous variables, CD for edges between continuous and discrete variables, and DD for edges between two discrete variables. Larger values of *λ* result in fewer edges in the output graph. CausalMGM assumes that continuous variables are normally distributed and are linearly related to one another and that categorical variables are multinomially distributed and can be modeled via a logistic regression. If the continuous variables are not normally distributed, we suggest users transform input variables using the non-paranormal transform in the *huge* R package (23).

#### StEPS

Since it is difficult for users to know which values to choose for each *λ* parameter, we provide an automated method to do so based on stability (19) At a high level, StEPS randomly draws subsamples of the dataset and learns an undirected graph using a fixed value of *λ* for all three edge types. It then computes the stability of the edges in the learned graphs across subsamples, and increases *λ* for those edge types that do not meet a stability threshold. This process is repeated until *λ* values are found for all three edge types. Due to the fact that many undirected models are learned, this process can be time consuming for large datasets, and should be used with caution.

#### PC-Stable

PC-Stable is a popular constraint-based method for causal discovery and is an order-independent extension of the PC algorithm (21). The inputs to PC-Stable are (i) a dataset, (ii) a starting graph and (iii) *α*, which is the *P*-value threshold for the conditional independence tests. This algorithm typically starts with a fully connected, undirected graph and performs conditional independence tests to remove edges between any two variables. First, unconditional independence tests are performed and an edge between *X* and *Y* is removed if *X* is independent of *Y*. The algorithm proceeds by increasing the size of the conditioning set and performing conditional independence tests for any remaining edges conditional on some subset of their neighbors in the resulting graph. This process continues until no more edges can be deleted in this manner. Then, the algorithm determines causal direction by (i) orienting colliders (i.e. a variable with two ‘parents’), (ii) avoiding the introduction of new colliders and (iii) avoiding directed cycles (loops) (21). In the CausalMGM web server, for increased speed and accuracy, PC-Stable starts with the undirected graph produced by CausalMGM instead of a fully connected graph. PC-Stable assumes that there are no feedback loops in the causal graph, that each variable is independent of its direct effects given its direct causes (causal Markov assumption), the conditional independence relations in the data come from applying the causal Markov assumption to the causal graph (causal faithfulness assumption), and that there are no unobserved confounders or selection bias in the data.

#### Independence test

PC-Stable requires an independence test suitable for the data. We have developed a regression-based independence test for mixed data types that we use with PC-Stable (7). Assume we are testing whether *X* is independent of *Y* given $\mathbf {Z}$. If *Y* is continuous, then we calculate the linear regression of *Y* using *X* and $\mathbf {Z}$ as predictors (if *X* is categorical, we use dummy-encoded binary predictors). The *P*-value of the test is then a *t*-test on the *β* coefficient of *X*. Alternatively, if *X* and *Y* are both categorical, then we perform a multinomial logistic regression predicting *Y* using *X* and $\mathbf {Z}$ along with a regression model using just $\mathbf {Z}$. The *P*-value for the test comes from a likelihood ratio test comparing these two nested models. By default in CausalMGM, the *α* threshold for this independence test is set at 0.05.

### Interactive visualization

**Figure 3.**
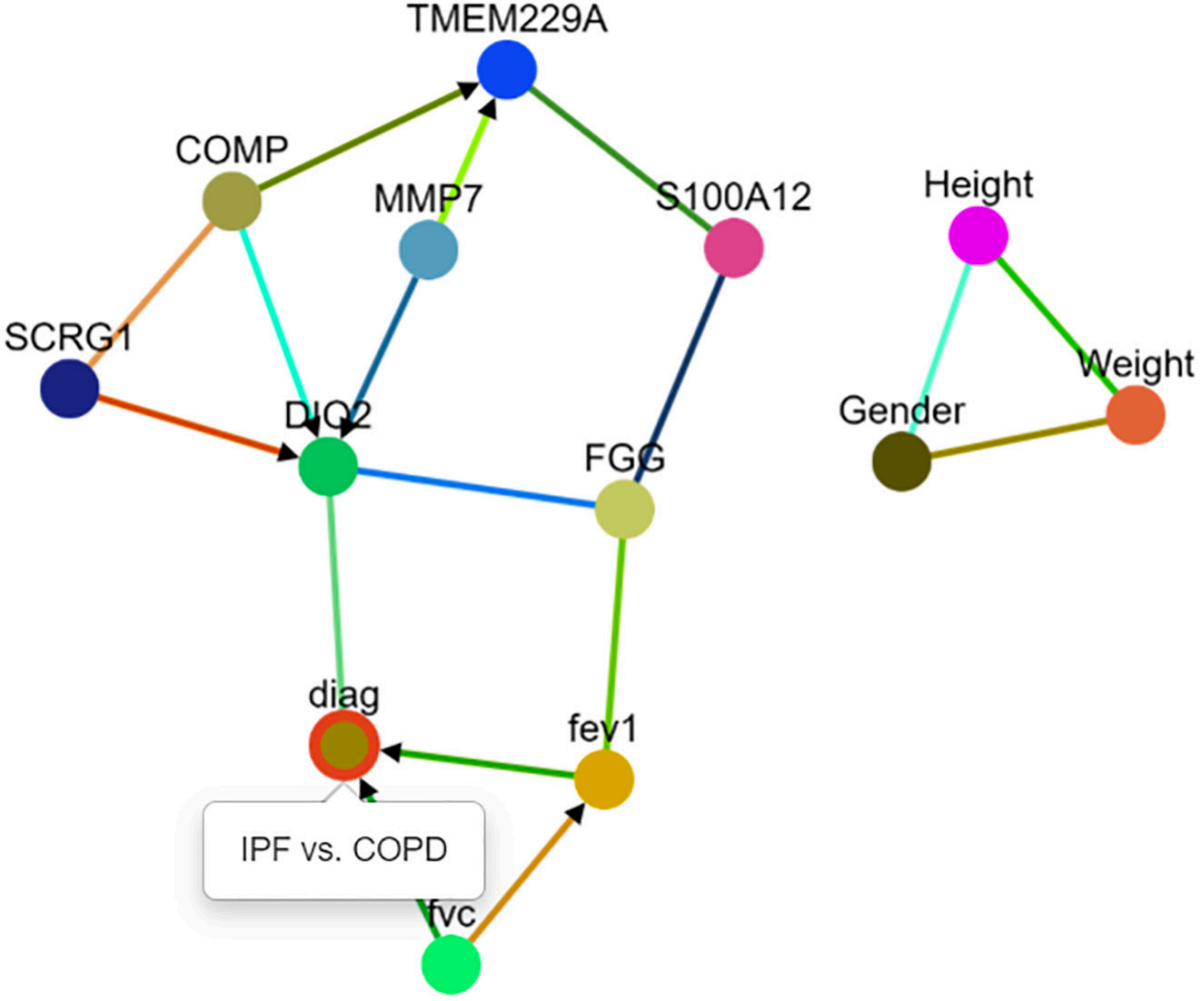
Learned causal graph from the sample dataset included in the web server. Arrows indicate cause-and-effect relationships and undirected edges indicate conditional dependence relationships.

As illustrated in Figure 3, the CausalMGM web server leverages the popular graph theory library Cytoscape.js to generate dynamic graphical representations that help researchers visualize the conditional dependency and causal relationships obtained from the previous steps. Here, the graphical output is an undirected (conditional dependence) or directed (causal) graph with vertices corresponding to variables and edges corresponding to dependencies between variables. This differs from protein–protein interaction or gene regulatory networks by modeling mixed data and by representing causal relationships from observational data alone. Our CausalMGM web server also provides the user with the option to annotate the nodes and edges inside each resulting graph. These visualizations can easily be downloaded into both SIF and JSON as formats, which allow the resulting graphs to be further analyzed using any desktop visualization tools such as Cytoscape.

## COMPUTATIONAL PERFORMANCE

To illustrate the efficiency of our CausalMGM web server, in Table 1 we provide a run-time analysis (in seconds) of each of CausalMGM’s main computation tasks (i.e. learning an undirected graph with MGM, learning a directed graph with PC-Stable, StEPS to find lambdas and Pref-Div for feature selection). We use five simulated datasets to cover a wide range of number of variables (50–500) and number of samples (500–20 000). Datasets were generated using the Lee and Hastie simulation method for mixed data [see also (24)]. We obtained these statistics on a server with an 18-Core Intel Core-i9 processor running at 2.6 GHz and 128 GB of main memory.

**Table 1.** Run-time of different variable and sample sizes

Variable size	Sample size	Learning task	Run-time (s)
50	500	Undirected graph	8.9
50	5000	Undirected graph	1649.7
50	20 000	Undirected graph	12 305
100	500	Undirected graph	9.35
500	500	Undirected graph	433.5
50	500	Directed graph	52.2
50	5000	Directed graph	16 832.3
50	20 000	Directed graph	16 243
100	500	Directed graph	67.7
500	500	Directed graph	1173.7
50	500	StEPS (find lambda parameters)	360.38
50	5000	StEPS (find lambda parameters)	6025.2
50	20 000	StEPS (find lambda parameters)	11 432
100	500	StEPS (find lambda parameters)	467.9
500	500	StEPS (find lambda parameters)	881.9
50	500	Pref-Div (feature selection)	0.54
50	5000	Pref-Div (feature selection)	2.7
50	20 000	Pref-Div (feature selection)	3.3
100	500	Pref-Div (feature selection)	1.2
500	500	Pref-Div (feature selection)	1.7

## USE CASE

Next, we present an example use case of running CausalMGM on a small dataset containing transcriptomic and clinical data. This dataset consists of RNA-Seq measurements for seven genes, seven clinical parameters and an outcome variable of diagnosis (‘diag’). The outcome is a binary variable for whether the individual had idiopathic pulmonary fibrosis (IPF) or chronic obstructive pulmonary disease (COPD). The goal is to identify genomic and clinical variables that distinguish these disorders, since the etiology of IPF remains unknown.

The sample dataset comes from a previous publication (19), in which the data were derived from the Lung Genomics Research Consortium. The dataset includes gene expression and clinical variables. For this dataset, the selected genes are those known to be important in one of two chronic pulmonary diseases (COPD and IPF) and the clinical features include age, gender and smoking history. The variable diag represents the final diagnosis (COPD or IPF).

For this experiment, we use the ‘find lambdas’ function to automatically identify the parameters of the undirected modeling, and we use PC-Stable to identify causal relationships with *α* = 0.05. We do not use feature selection for this dataset due to the small size. The result of the experiment is shown in Figure 3.

The learned model has some expected connections, such as those between gender, height and weight. One way to interpret these undirected edges is that they identify the ‘best predictors’ in the data. To predict weight on an unseen individual, the best variables to use are height and gender. The target variable of interest has two direct causes: forced expiratory volume in 1 s and forced vital capacity. These are two clinical tests that are commonly expressed as a ratio to indicate an individual’s overall lung function. The arrows indicate a causal relationship, meaning that the diagnosis made for an individual depends upon their lung function test outcome. Lastly, despite the fact that all of these genes are related to IPF and COPD, the model suggests that the only direct effect on diagnosis is DIO2; however, the edge is undirected meaning that this is not a causal effect. This implies that the changes in DIO2 expression may determine the disease status or vice versa or there is an unmeasured confounder causing both DIO2 and diagnosis. Since both IPF and COPD lungs are shown to have increased levels of DIO2 (25,26), this might advocate the presence of a confounder, which also impacts the differential diagnosis of IPF and COPD.

## DISCUSSION

In this work, we have presented the CausalMGM web server, which is designed for automated identification of cause-and-effect relationships from multi-modal, observational, biomedical data. We have described the details underlying the server implementation and shown a use case for how CausalMGM can improve data exploration. Future directions to improve CausalMGM could include measuring the uncertainty in the causal graphs, including methods to deal with hidden confounders, and allowing the user to encode their background knowledge and assumptions into the causal discovery methods. CausalMGM is a free service open to all non-commercial applications.
